# Gradually tapering off antipsychotics: lessons for practice from case studies and neurobiological principles

**DOI:** 10.1097/YCO.0000000000000940

**Published:** 2024-05-09

**Authors:** Mark A. Horowitz, Joanna Moncrieff

**Affiliations:** a Division of Psychiatry, University College London, Maple House, Fitzrovia, London^∗^; b North East London Foundation Trust, Goodmayes Hospital, Goodmayes, Ilford, UK

**Keywords:** antipsychotic withdrawal symptoms, dopaminergic hypersensitivity, gradual tapering, hyperbolic tapering

## Abstract

**Purpose of review:**

There has been an increasing focus on deprescribing in psychiatry recently, particularly of antipsychotic medication, with recognition that not all patients with psychotic disorders require lifelong medication. We summarize some empirical and theoretical papers, and examine case studies to provide instruction on this topic.

**Recent findings:**

Recent studies have found that slower tapering (over months or longer) of antipsychotics is associated with a lower relapse rate than quicker tapering (weeks). Case studies presented suggest that the process of reduction is associated with the precipitation or exacerbation of psychotic symptoms and that a slower process of reduction may minimize this effect. This may be because faster reductions cause greater disruption of homeostatic equilibria, provoking psychotic symptoms either as direct withdrawal symptoms or consequences of nonpsychotic withdrawal symptoms (e.g. insomnia) – although not all patients will experience withdrawal symptoms. This suggests that smaller dose reductions, especially at lower doses, made very gradually, may minimize the risk of psychotic symptoms.

**Summary:**

Slower tapering of antipsychotics may provide time for adaptations made to the presence of the medications to resolve, thus reducing the disruption to homeostatic equilibrium caused by dose reduction, potentially reducing the risk of relapse. Exacerbation of psychotic symptoms on antipsychotic reduction may not represent evidence of the need for a higher dose of antipsychotic on a long-term basis but may indicate the need for more gradual reduction. Gradual reduction of antipsychotics, especially after long-term use in clinical practice is prudent.

## INTRODUCTION

For decades, long-term ‘maintenance’ antipsychotic treatment has been the accepted norm for people diagnosed with schizophrenia and psychotic conditions. There is, however, increasing attention being paid to reducing and/or stopping antipsychotic medication in people with long-term psychotic conditions [1^▪^,2^▪^,^3^^▪▪^], with several large-scale randomized controlled trials on this subject taking place around the world and formation of a large international consortium of researchers around this issue (TAPER) [1^▪^,^4^]. This focus on reduction and cessation is due to recognition of the substantial adverse effects of this class of medication, that many patients wish to reduce medication because of unpleasant subjective effects, that their functioning may actually be improved in the long-term by dose minimization and that many clinicians are already incorporating this into their practice [2^▪^,^3^^▪▪^,^5^,^6^^▪▪^].

A 2020 systematic review of world-wide guidelines for the treatment of schizophrenia found that most currently recommend indefinite maintenance treatment and did not recommend antipsychotic discontinuation for multiple-episode schizophrenia [7]. On the other hand guidelines tended to shift over time from presenting reduction or discontinuation as ‘not recommended’ to ‘partially recommended’ for both ‘schizophrenia in general’ and first-episode schizophrenia [7]. All of the newer guidelines endorsed antipsychotic dose reduction strategies [7]. Some guidelines imply that treatment could be withdrawn after 1–2 years even in multiple episode psychotic conditions. For example, the NICE schizophrenia guidelines in the UK for treatment of subsequent episodes of psychosis recommends that clinicians ‘Inform the service user that there is a high risk of relapse if they stop medication in the next 1–2 years’, ‘If withdrawing antipsychotic medication, undertake gradually and monitor regularly for signs and symptoms of relapse’ and ‘After withdrawal from antipsychotic medication, continue monitoring for signs and symptoms of relapse for at least 2 years [8].’

The recommendations in these guidelines about maintenance treatment are derived from discontinuation studies, where patients established on antipsychotic medication are randomized to maintain or discontinue antipsychotics with relapse monitored for 1–2 years afterwards which find higher rates of relapse in the discontinued group [9,10^▪^]. However, these studies have been criticized because they involve rapid reductions of antipsychotics in the discontinuation group (mostly abruptly or over a few weeks) [11] meaning withdrawal phenomena might inflate the apparent rate of relapse in the discontinuation arm [1^▪^,2^▪^,10^▪^,^12^]. They also suffer from limited follow up periods and a narrow focus on symptom scores rather than outcomes which might be more important to patients [1^▪^]. It is therefore possible that the relapse prevention properties of maintenance treatment with antipsychotics may have been artificially inflated by the methodologies employed in these studies. One study showed that although the risk of relapse following discontinuation or reduction of antipsychotics was increased initially compared to maintenance treatment, in the long-term the risk equalized and people initially randomized to a reduction programme had improved social functioning [6^▪▪^].

Reflecting some of this uncertainty the authoritative Maudsley Prescribing Guidelines says ‘There are currently no evidence-based recommendations for antipsychotic withdrawal, but we suggest that it only be attempted in patients who have been in remission for 6 months (first episode) or 1 year (multi-episode) (p. 105) [13].’ Professor Sir Robin Murray parses this uncertainty by suggesting that ‘following recovery, the psychiatrist should work with each patient to decrease the dose to the lowest level compatible with freedom from troublesome psychotic symptoms; in a minority of patients this level will be zero [3^▪▪^]’. However, there are no specific guidelines for how to deprescribe antipsychotics [14], with the exception of an evidence- and consensus-based guideline on stopping clozapine [15], and a guide to stopping aripiprazole depot [16].

A substantial proportion of psychiatrists indicate a willingness to reduce antipsychotic medication in people who have experienced a single episode of psychosis, with a smaller but still sizeable proportion willing to reduce for people with multiple episodes of psychosis [17]. Taking into account the complexity, it has been suggested that all patients should have the opportunity to trial gradually discontinuing medication if that is what they want and if risks allow [2^▪^,^18^,^19^]. 

**Box 1:**
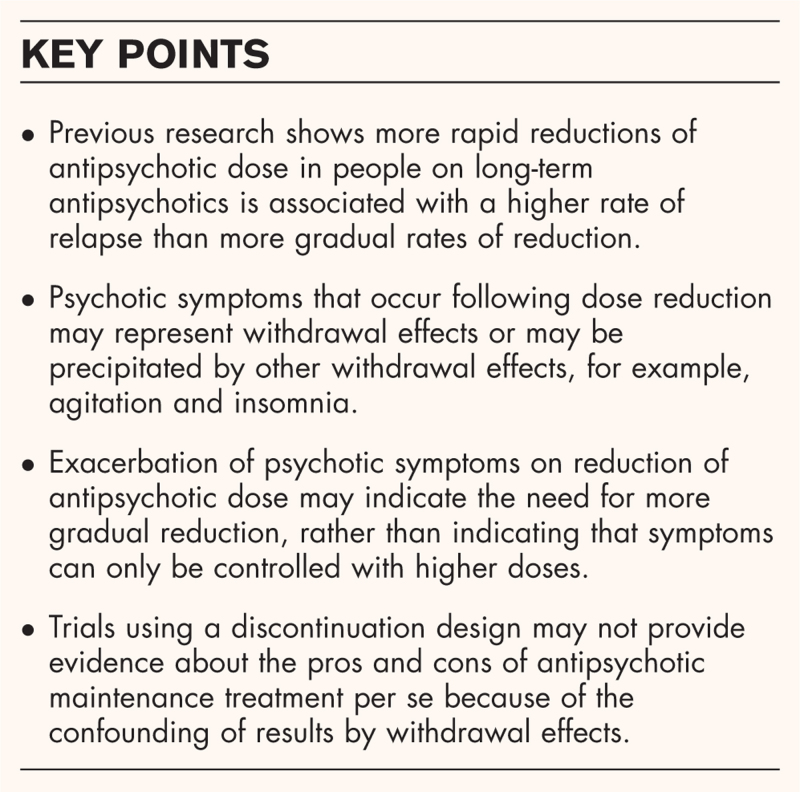
no caption available

## THE MANNER IN WHICH ANTIPSYCHOTICS ARE STOPPED

It is well established that gradual tapering of psychiatric medications, like benzodiazepines, produces a better outcome than more rapid tapering [20], and this principle is increasingly accepted for other classes of psychiatric drugs as well [21,22,23^▪^,^24^]. In parallel, there is recognition that the manner in which long-term antipsychotics are ceased may have some bearing on the rate of relapse [2^▪^,^25^^▪▪^,26^▪^,^27^,^28^]. There is general agreement that stopping long-term antipsychotics abruptly is likely to produce the worst outcomes for relapse [9,29^▪^] as evident from everyday clinical practice. Stopping antipsychotics more slowly may reduce the risk of relapse [25^▪▪^].

Stopping antipsychotics too quickly may provoke withdrawal effects, which may include psychotic symptoms per se, or may precipitate genuine relapse (often called withdrawal-associated relapse) possibly as a consequence of the occurrence of other withdrawal effects (such as insomnia, anxiety or agitation) [10^▪^,^25^^▪▪^]. Observation that people with no underlying psychotic disorder can experience psychotic symptoms, including cardinal symptoms of schizophrenia, after antipsychotics or related dopamine antagonists, initiated for reasons other than mental health conditions (e.g. nausea or trouble with lactation), are stopped abruptly suggests that antipsychotic withdrawal symptoms may include psychotic symptoms [10^▪^,^25^^▪▪^,30^▪^]. The clustering of relapses soon after the point of abrupt or quick discontinuation in discontinuation trials of antipsychotics in people with psychotic disorders [25^▪▪^,29^▪^], as compared to the more even distribution over time of relapses in the natural history of the disorder [25^▪▪^,^31^] is consistent with a withdrawal-related effect on symptom occurrence or relapse.

The role of the rate of reduction is evidenced in a meta-analysis that found that slower rates of taper were associated with lower overall rates of relapse (Table 1) [26^▪^]. This is consistent with an older meta-analysis that found lower relapse rates with more gradual dose reductions than quicker dose reductions [29^▪^]. An updated meta-analysis found evidence of an inverse gradient between risk of relapse and tapering period: abruptly stopping (2.42 relative risk (RR)); tapering over 1–10 weeks (2.28 RR); and tapering over more than 10 weeks (1.02 RR) [28]. This notion is also supported by an analysis that found that the relapse rate is five times higher for people stopping oral paliperidone compared with depot paliperidone even when observations are made when enough time has elapsed for wash-out of the depot [27,32] – indicating that rate of drug elimination (the only difference between the two conditions) was a key factor in determining overall relapse rate [16]. However, studies involving relatively gradual reduction still showed increased rates of relapse compared to maintenance treatment in the short to medium term [33,34] which might indicate that some people will experience a genuine re-emergence of their underlying condition during or after reduction and that not all will be affected by withdrawal effects or it may indicate that these reductions were not gradual enough.

**Table 1:** Relationship between duration of tapering and overall relapse rate in meta-analysis of antipsychotic discontinuation trials

Duration of tapering period	0 (abrupt)	1–2 weeks	3–10 weeks	>10 weeks
Relapse rate % (95% confidence interval)	77% (56–98%)	57% (35–80%)	47% (28–67%)	31% (26–36%)
Number of cohorts	14	12	7	10
Heterogeneity, *I*^2^ (%)	73	83	73	0

The significance between strata of tapering duration was *P* < 0.0001. Adapted from Bogers *et al.* (2020).

The RADAR study, which involved gradual reduction and discontinuation of antipsychotics in people with recurrent psychotic disorders, found similar rates of relapse and rehospitalization to discontinuation studies using much faster, often abrupt, reduction regimes [33]. Some commentators have concluded that the flexible, gradual taper employed in the RADAR study over up to 24 months precluded withdrawal effects [35], but some patients in the trial reported withdrawal effects [36^▪^] in interviews (though withdrawal effects were not measured quantitatively). Patient groups report that it can take many years after long term use of psychotropic medications (the average period of use in the RADAR trial was 17 years) to safely taper. [37^▪^], hence it is possible that tapering in the RADAR trial was too rapid. Moreover, as the tapering period was determined by individual clinicians they varied in their duration with some lasting several months but some only a few weeks [38]. Therefore some of the increase in relapse in the RADAR trial may have resulted from a too rapid taper. This notion is supported by the success of another randomized controlled trial which pursued an even slower rate of taper – 25% of the most recent dose every 6 months – which showed no difference in relapse rate compared with maintenance treatment [39], although no patients in this study discontinued medication completely, which may be another factor.

We provide some case studies to illustrate the experience of tapering patients off antipsychotics to shed more light on the process than can be discerned from aggregate RCT results. Following this we attempt to provide an explanation of the neurobiology underpinning these phenomena and draw out advice for clinicians in practice, as well as for research.

## CASE STUDIES

### Case study 1

Mr X, a 29 year-old man with a diagnosis of schizophrenia, characterized by auditory and visual hallucinations, and paranoid delusions had experienced his first episode of psychosis 8 years previously. He had residual auditory and visual hallucinations while on olanzapine 15 mg and aripiprazole 15 mg. He has not been employed or in education since his first episode. He was concerned that the medication was making him lethargic and unmotivated. He was tapered down to 5 mg of olanzapine and 5 mg of aripiprazole over the course of several months with no change to his mental state.

On reduction from 5 mg of olanzapine to 2.5 mg, the patient's auditory hallucinations increased and he experienced distressing visual hallucinations. He resumed taking 5 mg and these experiences resolved over the next two weeks.

Three months later he reduced his olanzapine to 3.75 mg (making three-quarters of a 5 mg tablet with a pill-cutter) with no noticeable change to his mental state. 3 months later he made a further reduction in olanzapine from 3.75 mg to 2.5 mg – he experienced a mild exacerbation of symptoms and returned to 5 mg for 3 days before returning to 2.5 mg on which he remained stable. His wife described his as ‘coming out of a fog’ and ‘I have my husband back’. He has enrolled in an electrician apprenticeship.

### Case study 2

Mrs Y, a 65 year-old lady with a diagnosis of schizophrenia was on long-term risperidone depot at a dose of 25 mg fortnightly. She disliked the sedation and emotional blunting produced by the medication and was concerned it was increasing her weight. She was switched from risperidone depot to the oral equivalent and then slowly reduced to 0.5 mg of risperidone over the course of 18 months. She experienced no significant change in her mental state over this period.

She then had her risperidone 0.5 mg ceased. A few months after this reduction her behaviour became more disorganized (she began throwing out her possessions) and she developed delusions that cars on the street were spying on her. The Home Treatment Team (HTT) were involved and increased her risperidone to 1 mg. The family were concerned at her behaviour and mental state and the HTT recommended increasing her dose to 2 mg of risperidone or re-commencing her depot at 25 mg fortnightly.

The patient was opposed to either an increase in dose or returning to the depot. Her family both wanted to respect her wish to be on less or no medication but wanted to see her well. As her risks were manageable (she was throwing out clothes and angry at parked cars, but continued to eat and sleep and live in the family house with several family members), she continued on 1 mg of risperidone.

Her symptoms did not resolve over the next 6 weeks and as the patient was to travel with the family overseas the HTT decided to impose a depot with the family in agreement. However, the patient refused the depot and travelled overseas with her family continuing 1 mg of risperidone a day. On return from their trip (after about 4 months) her mental state had returned to baseline, with a resolution of her paranoia regarding cars and she stopped throwing away possessions. The treating team, family and the patient were in agreement that she should continue on risperidone 1 mg and the patient was appreciative of experiencing fewer adverse effects.

### Case study 3

Mr Z, a 49 year-old male with a diagnosis of schizophrenia with residual tactile and auditory hallucinations was treated with 300 mg of melperone for several years. Melperone is an antipsychotic of the butyrophenone class, with a similar structure to haloperidol, but with a profile of receptor action more akin to atypical antipsychotics [40]. It is often used in treatment-resistant cases of schizophrenia where clozapine is not suitable [41] and its half-life is 3–4 h. The patient's weekly activities consisted of leaving the house to food shop twice a week. He found the drug made him tired during the day, and he had put on large amounts of weight.

His dose was reduced from 300 mg to 275 mg without incident. On reduction from 275 mg to 250 mg he experienced partial insomnia from the 2nd day to the 5th day afterwards associated with an increase in auditory hallucinations that were somewhat distressing. He decided to persist with the reduced dose and 1 week after the reduction his insomnia had resolved and his auditory hallucinations had returned to their baseline levels.

Given his experience in reducing from 275 mg to 250 mg he did not want to reduce from 250 mg to 225 mg, so we agreed to reduce his dose to 237.5 mg (by splitting a 25 mg tablet with a tablet cutter) 6 weeks after the last reduction. On doing so he experienced partial insomnia from 2nd to 4th day after reduction, associated with an increase in auditory and tactile hallucinations. He was provided with increased support via phone for this period.

Six weeks later he reduced his dose from 237.5 mg to 225 mg and 2 months following this to 212.5 mg. On each occasion he experienced 2–4 days of insomnia and an increase in auditory hallucinations that had resolved within a week after the reduction. He continued to make reductions of 12.5 mg every couple of months following this and reached a dose of 175 mg. He was provided with an extra phone contact in the week following dose reductions to reassure him, and became accustomed to the pattern of symptoms which occurred following dose reductions.

He increased his daily activities over this period, attending a day group for people with mental health conditions three times a week (lockdown permitting), had re-connected with an old friend and taken up bike riding. He lost 10 kg in weight. He reported that the improvement in his energy, and feeling better (’clearer in my head’) motivated him to persevere through the unpleasant symptoms that occurred whenever he reduced his dose.

## PSYCHOTIC SYMPTOMS AS WITHDRAWAL EFFECTS AND THE ROLE OF RATE IN TAPERING

These case studies demonstrate the possibility that withdrawal effects from antipsychotics include psychotic symptoms or that other withdrawal effects (e.g. insomnia) may precipitate them. In these case studies psychotic symptoms occurring after dose reduction faded over time without an increase in dose. This pattern of exacerbation and spontaneous resolution is consistent with a withdrawal effect, familiar from the reduction of other classes of psychiatric medication. These case studies also demonstrate the role of rate of tapering as a factor in determining risk of psychotic symptom exacerbation.

It has generally been thought that there is a threshold of pharmacological activity (e.g. dopamine D_2_ occupancy) above which antipsychotics are effective, with some individual variation between patients [42]. The process of establishing the minimum effective dose for an individual is then conceptualized as the process of reducing medication to the point at which symptoms emerge and then returning to a dose slightly above this to maintain symptom control. However, these case studies suggest an alternative interpretation – that the process of reduction itself can have a causal effect on psychotic symptoms and that psychotic symptoms may sometimes be a withdrawal effect or withdrawal-related effect. As a consequence, reducing dose more slowly can produce fewer psychotic symptoms than reducing more quickly – in this interpretation, rather than a threshold below which patients cannot reduce further it may be that more gradual reductions can avoid precipitating an increase in psychotic symptoms.

Case 3 in particular demonstrates a predictable pattern of exacerbation of psychotic symptoms following small dose reductions that spontaneously resolve over several days, suggesting that psychotic symptoms may be withdrawal effects from reduction of antipsychotics and may not necessarily represent unmasking of the underlying disorder. Case 1 demonstrates that the rate of reduction may be more important than the overall amount of the reduction. In both methods of reduction (either abruptly or staggered in two phases over 6 months) the overall dose reduction was 2.5 mg of olanzapine from 5 mg (equivalent to approximately 17 percentage points of D_2_ occupancy). However, in the second attempt at reduction performed over 6 months there was less provocation of psychotic symptoms. Case 2 demonstrates that there may be a considerable lag in re-establishing equilibrium to a lowered dose of medication and that psychotic symptoms may be the result of this disruption to the equilibrium. They may resolve without an increase in antipsychotic, but this may take a period of months.

## NEUROBIOLOGY UNDERPINNING SLOW TAPERING

An explanation of these phenomena whereby antipsychotic reduction is associated with a temporary onset or exacerbation of symptoms (i.e. consistent with a withdrawal effect) and that slower tapering can reduce this impact can be made by reference to the underlying neurobiology.

### Homeostatic adaptation to antipsychotic treatment

**FIGURE 1:**
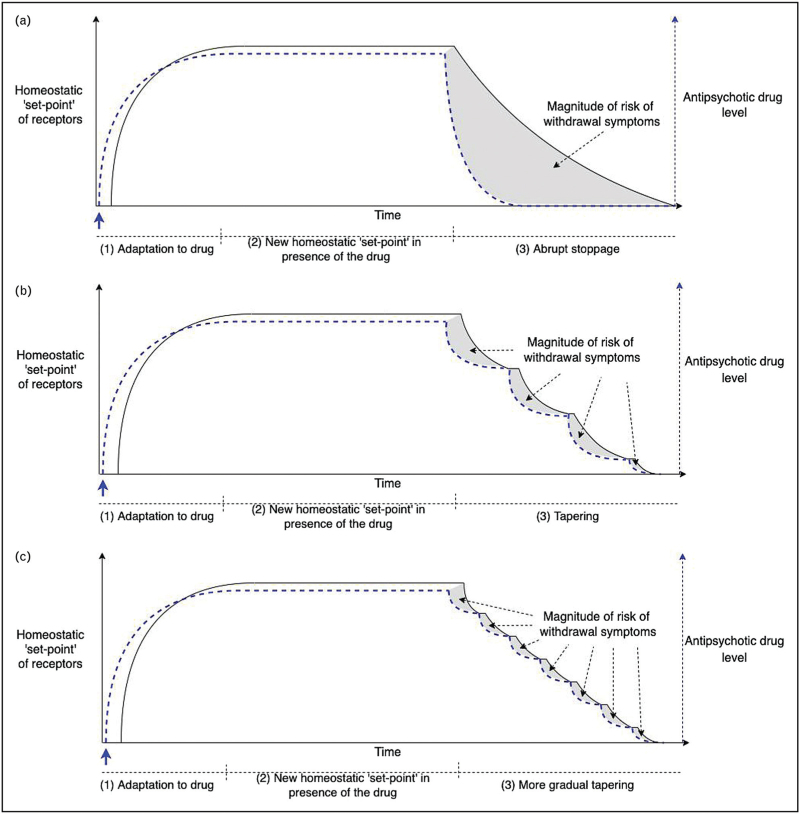
A conceptual model of how tapering antipsychotic medication affects the homeostatic equilibrium. Legend: This demonstrates the process by which the brain adapts to the presence and then removal of antipsychotic medication. The arrow represents the point at which an antipsychotic is introduced into the system, after which drug levels reach steady-state. (a) In (1) and (2), the homeostatic ‘set-point’ of the brain adapts to changes in available levels of synaptic neurotransmitters during long-term treatment with antipsychotics. In the case of dopamine, this will involve increased sensitivity to dopamine, often termed dopaminergic hypersensitivity or sometimes dopaminergic supersensitivity. (3) Abrupt reduction of antipsychotic will cause a rise in synaptic dopamine (and other neurotransmitters); re-adjustment of dopaminergic sensitivity will lag. Physiological levels of synaptic dopamine will be experienced as excess by the system adjusted to lower levels of dopamine. This excess may produce similar consequences to dopaminergic agonists including psychotic symptoms. When dopaminergic sensitivity re-adjusts to new higher levels of dopamine the effects of excessive dopamine should resolve. Similar principles should apply to other neurotransmitters, including, for example, cholinergic rebound on reduction of medications with pronounced anticholinergic effects. (b) Smaller reductions in dose in (3), should cause lesser disruption to the homeostatic equilibrium, provoking less symptoms, including potentially psychotic symptoms. If enough time is allowed to elapse between dose reductions then the system will have enough time to re-equilibrate to higher levels of dopamine before the next dose reduction is made. (c) Even smaller reductions in dose may further minimize the consequences of dose reduction. Source: Adapted from Horowitz and Taylor (2021) [54].

Long-term treatment with antipsychotics causes adaptations to the presence of the drug which seek to minimize their effects on the brain according to the process of homeostasis (Fig. 1) [43]. The most recognized adaptation to the dopamine D_2_ blockade of many antipsychotics is increased D_2_ sensitivity [25^▪▪^,^44^]. The nature of adaptation made to the antipsychotic will vary depending on the receptor targets of the medication: for example, drugs with strong anticholinergic effects, such as clozapine, are likely to produce up-regulation of cholinergic receptors [45]. There will be similar homeostatic responses to other targets of antipsychotics with up-regulation of target receptors occurring in the presence of antagonist effects, and down-regulation in the instance of agonist effects.

There is empirical evidence of increased D_2_ sensitivity in response to antipsychotics. In rodents, 9 months of haloperidol treatment led to a two- to threefold increase in D_2_ receptors, which stayed elevated for at least 2 months following haloperidol withdrawal [46], a time period equivalent to more than a year in humans [47]. In clinical subjects, meta-analysis of molecular imaging studies in people finds an increase in D_2_/D_3_ dopaminergic sensitivity in people who had been exposed to antipsychotics and not in antipsychotic-naïve patients [48]. One study quantified this increase as 30% relative to antipsychotic-naïve patients [49].

### Reducing the dose of antipsychotics – disruption of equilibrium

Long-term treatment with antipsychotics will produce an equilibrium at a new homeostatic ‘set-point’ (Fig. 1). When the dose of a drug is reduced it will lead to a ‘mismatch’ between the level of neurotransmitter available and the adjusted sensitivity of the receptor. Taking dopamine as an important and representative example: when the dose of a chronically administered antipsychotic is reduced the level of synaptic dopamine will rise. The D_2_ receptors in this system will be adapted to lower levels of dopamine (after long periods of blockade) and so this rise will be experienced as a relative excess of dopamine. The consequence of this excess of dopamine may be equivalent to a patient being exposed to a dopamine agonist (e.g., an amphetamine) and produce similar effects: agitation, insomnia and potentially psychotic symptoms [50].

According to homeostatic principles D_2_ sensitivity will re-adjust to increasing level of synaptic dopamine (the reverse of the initial adaptation to lowered levels of dopamine when the drugs were commenced); that is, the sensitivity of dopaminergic receptors will reduce. The time required for this process of re-adaptation is currently poorly understood. From animal studies after 9 months of antipsychotic administration (years in human-equivalent terms) [47], it can take more than a year (in human equivalent time) for this dopaminergic sensitivity to reduce [46]. Upon re-adaptation of receptors to higher level of dopamine (or other neurotransmitter perturbations consequent to lowering antipsychotic dose) the effects of excessive dopamine (according to the homeostatic ‘set-point’ of the system), including psychotic symptoms, would be expected to resolve.

If perturbations to the system were reduced – that is by making smaller reductions spaced out at greater intervals (tapering), allowing the system to re-adapt to increased levels of dopamine before making the next dose reduction – the likelihood (and magnitude) of withdrawal effects (or their downstream consequence), including psychotic symptoms or relapse would be expected to be reduced (Fig. 1b and 1c) [25^▪▪^].

## INTERPRETATION OF CASE STUDIES

The cases outlined above are consistent with this explanation. In Case 1 the same-sized reduction in dose from 5 mg to 2.5 mg of olanzapine when made abruptly caused psychotic symptoms whereas when it was performed in two separate steps with 3 months in between each step it provoked less intense psychotic symptoms. This suggests it was not being *at* a dose of 2.5 mg that produced the emergence of psychotic symptoms (i.e. being below some threshold of D_2_ blockade) but rather *the rate of reduction* in getting to this dose played a role in provoking psychotic symptoms. As in Fig. 1a, an abrupt reduction in input from the drug would cause a large ‘mis-match’ between the level of synaptic dopamine (or other neurotransmitter affected by olanzapine) and that ‘expected’ by the receptor (its homeostatic ‘set-point’). This would produce an apparent excess of dopaminergic stimulation of sensitized receptors, which might explain the exacerbation of psychotic symptoms in the patient.

In contrast, as in Fig. 1b, reduction in smaller steps would have produced a mis-match between expected and provided inputs but to a lesser degree. The delay before further reductions would enable the system to adjust to higher levels of synaptic dopamine: this would prevent super-imposition of withdrawal effects from rapidly made reductions. Indeed, as in Fig. 1c, making even smaller reductions spread out over time may further minimize the disruption caused to the equilibrium. It should be noted that because of the hyperbolic relationship between dose of antipsychotic and its effect on receptor targets, according to the law of mass action [51], including but not limited to dopamine blockade, that as total dose gets lower smaller decrements of dose will be required to produce the same rate of reduction of receptor occupancy and avoid causing a greater degree of disruption to the homeostatic equilibrium [25^▪▪^,^52^].

Case 2 is consistent with the interpretation that psychotic symptoms may be part of the withdrawal syndrome from antipsychotics and that they might resolve spontaneously without an increase in dose. This patient experienced an exacerbation of psychotic symptoms potentially provoked by a too rapid decrease in antipsychotic dose. The abrupt reduction from 0.5 mg of risperidone to 0 mg, causes a reduction of 29.4 percentage points of D_2_ occupancy, was larger than the reduction in occupancy caused by reducing from 4 mg to 1 mg (27.2 percentage points) staggered over many months [52,53]. After an increase to 1 mg of risperidone these symptoms resolved but the process took months, perhaps indicating the gradual nature of neuro-adaptation to lower levels of antipsychotics.

Case 3 emphasizes the possibility that exacerbation of psychotic symptoms may be a withdrawal effect. Small reductions in melperone produced a predictable pattern of psychotic symptom exacerbation with onset a few days after dose reduction, resolving several days later with no change in medication dose. This suggests that these symptoms (insomnia, psychotic symptoms) were withdrawal symptoms, which self-resolved when the patient's brain had re-adapted to a higher level of neurotransmitter, following removal of blockade, similar to Fig. 1c. This pattern of onset, peak and resolution seems quite distinct from the notion of a threshold dose below which psychotic symptoms would be produced and is more consistent with a withdrawal effect. Had much larger reductions in antipsychotic dose been made then the large increase in psychotic symptoms may simply have been interpreted as a relapse of the patient's underlying condition because there would be no opportunity to observe their resolution over time as the consequent effect on behaviour and mental state might have necessitated more assertive intervention. This may be what is observed in the emergency department after abrupt discontinuation of antipsychotics by patients in some cases.

## ANALOGY

Connections might be drawn to reducing the dose of other psychotropic medications [54]. For example, reducing benzodiazepines at too great a rate can produce unpleasant withdrawal symptoms such as anxiety, insomnia or agitation (which risk being mis-interpreted as re-emergence of an underlying condition) [55]. Indeed, rapid reduction of benzodiazepines can in some cases precipitate psychotic symptoms, including hallucinations, in people with no preexisting history of psychotic disorders [56]. Slowing the rate of tapering can produce more tolerable withdrawal symptoms that are lesser in intensity and resolve over a shorter time period [55].

An analogy may be made to ‘the bends’ experienced by scuba divers when rising to the surface too quickly after a deep dive. Their bodies have adapted to higher air pressure whilst diving; on rising to the surface of the water too quickly they experience a drop in pressure that produces too great a degree of a disruption to their established equilibrium, manifesting as uncomfortable symptoms called ‘the bends’. The condition is prevented by slowing rising to the surface over time, allowing time for the body to adapt to the new pressure conditions. The faster one rises the more likely ‘the bends’ are to occur. Treatment involves recompression that is, an increase in pressure and then a slow titration to sea level pressure based on the person's response. Similarly, for antipsychotic discontinuation the more abrupt the change in level of drug the more pronounced the symptoms – and if symptoms are too severe then re-instatement of a slightly higher dose (equivalent to re-compression) and subsequent more gradual reduction of the dose more carefully may minimize further symptoms. An ounce of prevention is also worth a pound of cure because once significant disruption to the system is produced it may be more difficult to reverse it, as with the protracted withdrawal syndromes, noted for benzodiazepines, antidepressants, and other psychiatric drug classes, including antipsychotics [57,58^▪^].

## CONCLUSION

### For research

The emergence of psychotic symptoms that register as relapse on symptoms scales in discontinuation trials of antipsychotics may not always represent re-emergence of the underlying disorder in the absence of a protective dose of medication but may sometimes be the consequence of too rapid reduction of medication leading to withdrawal symptoms, including psychotic symptoms or withdrawal-associated relapse (genuine relapse precipitated by withdrawal-associated de-stabilization) [2^▪^,^9^,10^▪^]. Of course, not all patients will experience withdrawal effects on reducing or stopping their dose of antipsychotic and some will experience a genuine relapse. Nevertheless, the possibility of withdrawal confounding the detection of relapse should prompt us to be cautious in interpretation of discontinuation trials of antipsychotics in which antipsychotics are often stopped over weeks or depots stopped abruptly [11,12,32,59] (which may not be slow enough to allow re-adaptation of receptors to changed levels of available neurotransmitters). This may also explain why the relapse rates in the maintenance arm and discontinuation arms of antipsychotics discontinuation studies tend to converge at about three years – as was evident in the MEFISTOS trial [6^▪▪^] and a meta-regression of 65 antipsychotic discontinuation trials [11]. This time period may reflect the time taken for receptor sensitivity to re-adapt to higher levels of dopamine (and other neurotransmitters) after long-term treatment is stopped and for exacerbation of symptoms to resolve.

Future studies should carefully consider the rate of tapering in discontinuation trials as a potential confounder and seek to minimize this confounding by employing gradual tapering which may need to be over years for people on long-term treatment. It is possible that even slower tapering than that employed in the RADAR trial (12–18 months) [33] may be needed for people who have been taking antipsychotics for very long periods.

### For clinical practice

Finding the minimum necessary dose of antipsychotics may not be as simple as finding the threshold below which a patient might have psychotic symptoms [3^▪▪^] but rather may involve finding a rate of reduction which causes either no discernible de-stabilization or a degree of de-stabilization that is tolerable within the circumstances of the patient. The degree of de-stabilization that is tolerable may be dependent on the nature of the patients’ condition, the degree of risk involved and the social support available [9].

This suggests that if a patient experiences psychotic symptoms on reduction of their dose of antipsychotic that this should not lead to the automatic conclusion that they require a higher dose of antipsychotic on a long-term basis, but rather that they may simply need to have reduced their dose more gradually – that is by smaller decrements and/or spaced out at greater time intervals. When an increase in psychotic symptoms does occur in the process of tapering the clinician has two options (ideally sharing the decision making with the patient, either through an agreed upon contract before the process of tapering or during the process): if the symptoms are not overly disruptive, and risk is not concerning, it may be possible to allow more time for symptoms to resolve (perhaps with greater support put in place during this period). Alternatively, a small increase in dose may be wise to allow re-stabilization to occur more quickly; and thereafter to reduce at a more gradual pace. Notably, stabilization on a lower dose or after a small increase in dose may take months (not days or weeks) because of the time taken for the brain to re-adapt to lowered levels of drug action (see Case 2).

It therefore may be prudent to proceed with reductions cautiously and gradually from the beginning of a taper so as not to produce unpleasant disruption which may cause anxiety for the patient and those around them. This approach to tapering antipsychotics shares the same principles as recommended in guidance for tapering other psychiatric medications, including antidepressants and benzodiazepines: that is, titrating the rate of reduction to what is tolerable for the patient [22] and may need to be as slow as reductions of 5-10% of the most recent dose per month (so that reductions become smaller and smaller as the total dose gets lower).

The rate of reduction requires striking a balance between harm caused by ongoing exposure to the medication and harm caused by too rapid reduction, a balance that will vary for each individual. Notably, reductions will probably need to be made by smaller and smaller decrements as total dose gets lower because of the hyperbolic relationship between dose and receptor occupancy [15,52].

Given the evidence presented above highlighting the convergence in relapse rates between the maintenance and discontinuation arms in antipsychotic discontinuation trials at three years, this suggests that tapering patients over approximately three years (although with substantial individual variability) may be required in order to minimize the risk of significant de-stabilization in people stopping long-term antipsychotics [25^▪▪^]. Notably, as the degree of adaptation may be less for people treated short-term with antipsychotics it may be possible to stop drugs more quickly in these patients [2^▪^]. This is consistent with recent studies that found that a majority of patients with schizophrenia on long-term treatment were able to reduce their dose (in a hyperbolic pattern) by on average 40% over two years without an increase in relapse compared to maintenance treatment [60^▪^] and that reducing dose by 42% over 6 months (in a small sample) produced no increase in relapse compared with patients maintained on their antipsychotic [61].

## Acknowledgements

*We thank Adele Framer for help preparing the images inFig. 1*.

*Ethical approval: Informed consent was obtained from all three patients presented as case studies.*

### Financial support and sponsorship

*No specific funding was received for this work. MH is supported by a clinical research fellowship at North East London NHS Foundation Trust (NELFT). This funding source had no role in the writing of the manuscript or the decision to submit it for publication.*

### Conflicts of interest

*M.A.H. reports that he works as a Clinical Research Fellow on the NIHR-funded RADAR study examining reduction and discontinuation of antipsychotic medication in people with psychotic disorders. He is an unpaid associate of the International Institute of Psychiatric Drug Withdrawal and a member of the Tapering Antipsychotics and Evaluating Recovery (TAPER) group consisting of international psychiatric researchers. He is co-founder and a consultant to Outro Health, a digital clinic in North America which helps people to safely stop unnecessary antidepressants through supported, gradual, hyperbolic tapering. He has received honoraria for lectures to universities and hospitals on safe deprescribing of psychiatric medications, including the University of Washington. JM reports grants from the National Institute of Health Research for the RADAR trial, examining reduction and discontinuation of antipsychotic medication in people with psychotic disorders, that she is co-chairperson of the Critical Psychiatry Network (an informal group of psychiatrists) and a board member of the unfunded organization, the Council for Evidence-based Psychiatry. Both are unpaid positions. She receives royalties for several books about psychiatric medications.*

## REFERENCES AND RECOMMENDED READING

Papers of particular interest, published within the annual period of review, have been highlighted as:

▪ of special interest
▪▪ of outstanding interest
